# Medico-Legal

**Published:** 1909-12

**Authors:** John A. Rafter


### PROGRESS IN MEDICAL SCIENCE.

### Medico-Legal

Conducted by JOHN A. RAFTER, M. D.

### THE MEDICAL EXPERT.

The court and jury must obtain certain information from medical science in order to arrive at just conclusions and in no other way can they obtain such information, than from the medical expert. The court decides upon the qualifications of the expert, and he is put on the stand and subjected to a cross-examination, intending to break down the opinion which the court has decided he is competent to give. This makes the expert appear ridiculous and renders his testimony valueless for court and jury. Surely this is not the fault of the medical expert but it is the fault of the system of the present state of the law as administered.

It has become somewhat the fashion for lawyers and judges to cast most objectionable reflections upon the medical expert. There are some notable exceptions to this position by certain members of the bar and bench. The accusations and criticisms are in my judgment most unjust. That there are defects in the present conditions no one will deny, but the trouble comes from the antiquated and faulty system in vogue in the courts and not from the medical expert, with few exceptions. The time has arrived when, with the present knowledge in medical science, the medical expert should no more be considered a witness to be placed on the stand and cross-examined after his qualifications have been accepted by the court, than a judge should be so considered when called upon to interpret a point of law.

An expert is sworn to tell the truth, the whole truth, and nothing but the truth. When he comes to be cross-examined he is not permitted to tell the whole truth. If he attempts to explain his position in order to make his testimony clear, he is brought up with a sharp turn and informed that he is not to argue the case, and must answer the question by yes or no; something he cannot do and state the information he possesses. A lawyer skilled in cross-examinations, can so frame his questions that an expert is obliged to give information entirely foreign from what he desires. At least the lawyer can so put his question that the answers of the expert is obliged to give will make an impression on the court and jury entirely different from what the expert intended. This is not justice. The expert is placed in a false position and it is no fault of his. There are many other features of the present system equally objectionable and should be corrected and it is gross injustice to charge the expert with giving testimony that is worse than useless.

It has been my lot, not one of my choosing either, to have been an expert witness in quite a number of murder cases, in my state. Frequently, I have been retained—so to speak—by one side and upon examination of the prisoner, and after hearing all the testimony on both sides, gave opinion favorable to the other side. I have always adhered to the right to withhold my opinion—positively declining to express an opinion—until I have all the facts bearing on the case. The hypothetical question as usually framed and put, has frequently been a bar to a correct medical opinion and a source of embarrassment to a conscientious witness. —Dr. Drewry.

Until the difference of opinion as to what constitutes insanity, as viewed by the medical and legal professions, has been brought closer together, there will continue to be debate without satisfactory conclusion as to the province of the insanity expert in médico-legal cases. While the battle wages, the public stands aghast at the ingenuity of lawyers, the scientific evidence of the alienist-experts and the increasing number of criminals who get off on the insanity plea. The lawyer on the one hand gets roundly abused for his shrewdness, and the medical witness on the other hand, is criticised for his expert opinion of the client's mental disease and consequent irresponsibility.

Every precaution should be exercised to prevent the insanity plea from being abused and thereby giving the real criminal undeserved protection, nevertheless, it is entirely in line of modern progress in forensic medicine, to have every one charged with crime in which there is a doubt about his mental condition examined by fair and competent experts. This matter is of such vital importance that our laws and our practices should be amended, so to put it, in the power of the court to call the experts instead of having them called by the defense or defend prosecution. The witness so-called, should of course be subjected to cross-examination by both sides in the case. This would at least prevent the possibility of the expert being a partisan or an advocate which he should never be. Another plan, a modification of the above, is to have the court appoint a commission of experts whose duty it would be to examine and study the case in question and report its opinion to the court. The report should be open to both the prosecution and the defense and if desired used in evidence.

It is time that the medical and legal professions and the courts of this country were taking an advanced position in the matter of expert testimony, for it cannot be controverted that in many places it is in a bad way and is not respected as it should be. This is largely due to the inefficiency and the questionable character of physicians too often employed, and the prevalent system of proving insanity rather than ascertaining the real mental condition. We are nearly half a century behind some other countries in this department of medical jurisprudence.

—*Medico-Legal Journal.*

